# Neurology Trainees' Perceptions of the Educational Value of the Grand Round: A Qualitative Focus Group Study

**DOI:** 10.7759/cureus.113684

**Published:** 2026-07-30

**Authors:** Safia Lakehal, Guleed Adan

**Affiliations:** 1 Intensive Care Unit, Northern Care Alliance NHS Foundation Trust, Manchester, GBR; 2 Neurology, Northern Care Alliance NHS Foundation Trust, Manchester, GBR

**Keywords:** grand rounds, neurology education, postgraduate medical education, psychological safety, qualitiative research

## Abstract

Background

Grand rounds remain an important educational forum within academic medical centres and a longstanding component of postgraduate medical education. At the study centre, the weekly neurology grand round represented the principal recurring organised teaching session available to neurology trainees. Its educational value has not previously been formally explored from the perspective of trainees. Educational commentaries have raised concerns regarding increasing reliance on didactic teaching formats and the impact of public questioning on psychological safety and learning. This study therefore aimed to explore neurology trainees' perceptions of the educational value of the grand round, identify factors influencing its educational value, and examine whether these perceptions varied according to trainee seniority.

Methods

A qualitative study using semi-structured focus groups was conducted at a tertiary neuroscience centre in North West England. All eligible neurology trainees within the regional training programme were invited to participate using a purposive sampling strategy. Eight trainees participated across two focus groups. Discussions were audio-recorded, transcribed verbatim, and analysed using an inductive conventional content analysis approach. Two reviewers independently coded the transcripts before reaching consensus on higher-order categories and subcategories, which are presented as themes and subthemes.

Results

Eight neurology trainees participated across two focus groups. Five themes were identified: educational role of consultants, questioning style, learning format, learning environment and trainee anxiety, and the role of time and experience. Participants valued the case-based format of grand rounds, particularly its ability to contextualise theoretical knowledge and provide insight into consultant-level clinical reasoning and management decisions. Consultant involvement was perceived as an important educational aspect of the grand round. However, participants also identified barriers to learning, including anxiety associated with the learning environment, broad open-ended questioning, and repeated targeting of individual trainees during public questioning. Perceptions varied according to trainee seniority, with junior trainees reporting greater anxiety and reduced engagement, whereas senior trainees increasingly valued grand rounds as preparation for independent consultant practice.

Conclusion

Neurology trainees viewed the grand round as a valued educational activity, particularly for contextualising theoretical knowledge and observing consultant-level clinical reasoning and management decision-making. However, trainees perceived the educational value of grand rounds to be influenced by questioning style, psychological safety, and trainee seniority. Greater consideration of these factors may help optimise the perceived educational value of grand rounds. Further evaluation in other neuroscience centres would help determine the transferability of these findings.

## Introduction

Neurology training in the UK comprises five years of dedicated speciality training, leading to the award of a Certificate of Completion of Training [1]. Grand rounds represent an important educational forum within academic medical centres [2] and remain a longstanding component of postgraduate medical education [3]. At the study centre, the weekly neurology grand round represented the principal recurring organised teaching session available to neurology trainees. Consequently, a substantial proportion of trainees' formal learning and preparation for independent practice was concentrated within this session, making its educational value an important focus for evaluation.

Grand rounds are intended to educate trainees, disseminate knowledge, and provide updates in diagnosis and treatment [3]. They have also been recognised as advancing clinical knowledge and facilitating the development of clinical reasoning skills [4]. 

The broad structure of the grand round, comprising discussion of a clinical case before an audience of trainees, consultants, and students, descends from the case-based traditions of Charcot and remains widely recognisable across UK neuroscience centres today [5]. The case-based format of the grand round uses authentic clinical cases to integrate theoretical knowledge with clinical practice [6], an approach that has been associated with positive learning outcomes in medical education [7].

Yet aspects of the modern grand round have been the subject of debate within the literature. Published commentaries have raised concerns regarding an increasing reliance on PowerPoint (Microsoft Corp., Redmond, WA) [8], limited use of evidence-based educational methods [9], and reduced engagement and educational value [8]. This sentiment was reflected by the American physician Lawrence Altman, who famously remarked, “grand rounds are not so grand anymore” [10].

Separately, educational commentaries have raised concerns regarding the public questioning of trainees before senior colleagues, a practice that has been likened to "pimping" [11]. This approach has also been criticised for its potential to generate discomfort and reinforce hierarchy at the expense of psychological safety and learning [12]. These published critiques have contributed to ongoing debate regarding the educational role of this central component of neurology training.

The educational value of grand rounds can be understood through several established educational theories, including the principles of adult learning and the relationship between the learning environment and motivation [13-14]. What is less known is how neurology trainees themselves perceive the educational value of the grand round. 

Despite its longstanding role in postgraduate medical education [3] and its central place within the study centre's training programme, the neurology grand round has not previously been subject to formal appraisal in the literature, and the perceptions of the trainees for whom it is principally intended remain undocumented.

This gap matters because the educational value of a learning activity cannot be assumed from its format alone; it depends on how the intended learners experience it, and on the barriers and contributors to learning they encounter in practice. The educational commentaries describing didactic drift [8] and the climate of public questioning [11] raise important questions about aspects of contemporary grand rounds that can only be confirmed, refuted, or contextualised by exploring trainees' experiences and perceptions directly. Without this understanding, efforts to optimise the educational benefit of the grand round risk being misdirected. This study therefore aimed to explore neurology trainees' perceptions of the educational value of the grand round. Specifically, it sought to identify the factors trainees perceived as positively and negatively influencing that value, and to examine whether these perceptions changed as they progressed through their training.

## Materials and methods

Study design and theoretical approach

This qualitative study explored neurology trainees’ perceptions of the educational value of grand rounds within a large tertiary neuroscience centre. Qualitative inquiry through focus groups was selected as it offered participants a platform to express their views in their own terms, allowing for more insightful data to be collected than would have been possible with quantitative methods [15].

Data from focus groups were analysed using a conventional content analysis approach, with this approach being most appropriate when describing a relatively understudied phenomenon, in this case the perception of neurology trainees. With little prior theory existing, the inductive approach allowed codes to be derived from the data itself rather than from a pre-existing set of categories, ultimately helping to keep the findings data-driven.

Setting

The study was conducted at the Walton Centre for Neurology and Neurosurgery, a tertiary neuroscience centre in the North West of England. At the time of the study, the host neuroscience trust employed approximately 15 specialist neurology trainees, distributed across five to six hospitals throughout the region. All trainees and consultants attend the host centre on the day of the weekly neurology grand round, unless on pre-approved leave. The grand round at this tertiary centre follows a traditional case-based format in which two trainees each present a clinical case, after which a chairing consultant questions trainees seated in the audience about the clinical content. The audience also includes consultants and trainees from allied neuroscience specialities, more junior medical trainees, and medical students.

Sampling and recruitment

The study population comprised all neurology specialty trainees within the North West regional training programme. A total population sampling approach was used, a form of purposive sampling in which all members of the eligible population sharing the characteristic of interest are invited to participate. This was because they had direct experience of attending regional neurology grand rounds and were therefore well placed to provide rich, relevant insights into the educational phenomenon under study.

Inclusion criteria were enrolment as a neurology speciality trainee in the North West region and active participation in clinical training during the study period. At the time of the study, there were 15 trainees distributed across six hospitals. Exclusion criteria were prolonged absence from clinical training, including parental leave, sickness absence, or out-of-programme research, as it would not have been reasonable to expect these trainees to engage with trust emails whilst away from training. Five trainees met these exclusion criteria and were therefore not invited to participate. Trainees who had completed their Certificate of Completion of Training (CCT) but had not yet commenced a consultant post were also excluded, as they had completed the training programme and were no longer considered trainees. No individuals met this criterion. Following application of the inclusion and exclusion criteria, 10 eligible trainees were invited to participate in the study. The inclusion and exclusion criteria are summarised in Table 1.

**Table 1:** Inclusion and exclusion criteria for study participation

Inclusion criteria	Exclusion criteria
Neurology speciality trainees in the North West region	Prolonged absence from training during the study period: long-term sickness, parental leave, out-of-programme research
Trainees actively participating in training during the study period	Trainees who had completed their Certificate of Completion of Training (CCT) but had not yet started a consultant post

**Figure 1:**
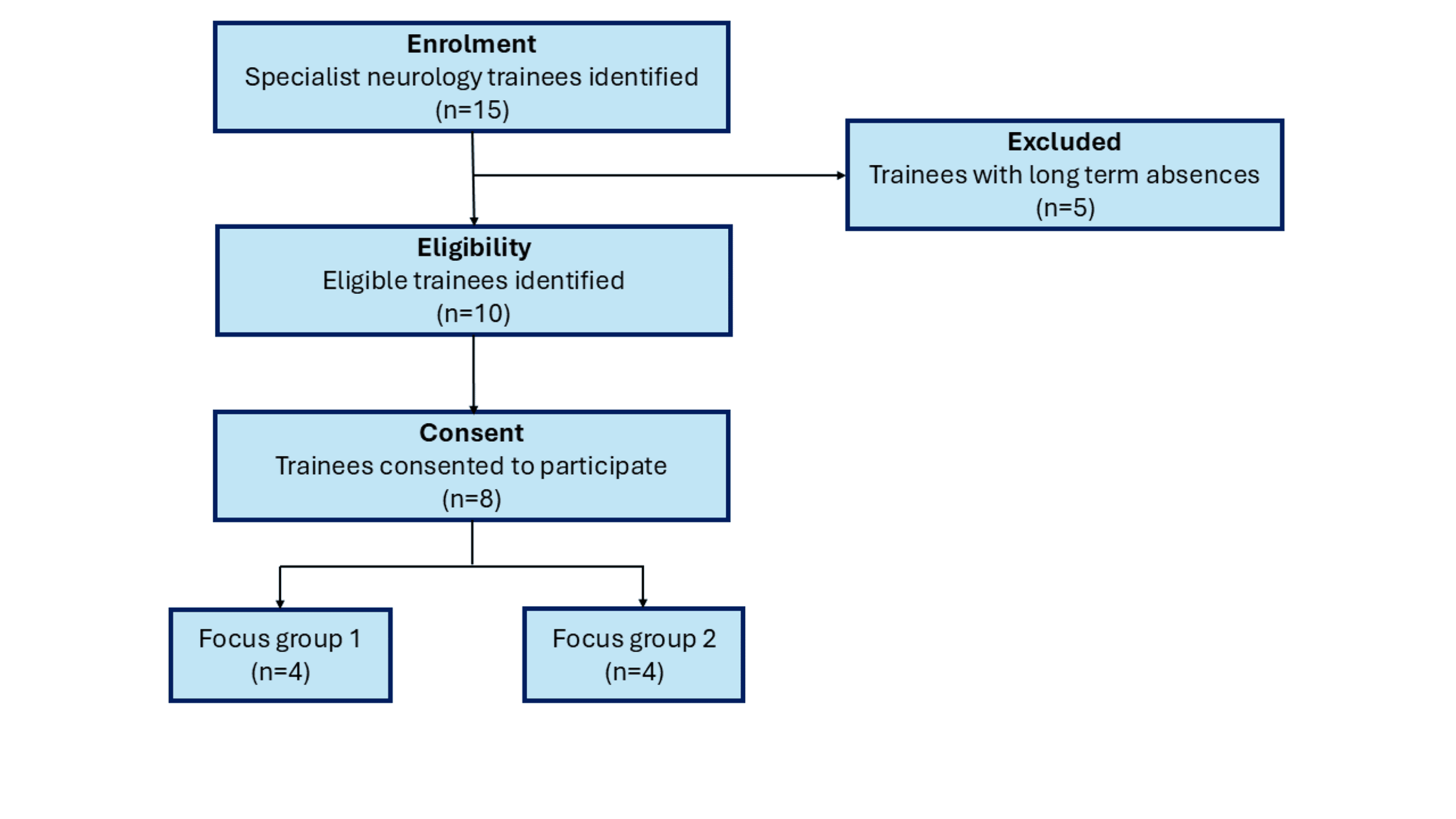
Study enrolment and participant flow Flow diagram showing participant recruitment, exclusions and allocation into focus groups. n = number of participants.

To reduce the potential for self-selection bias and to obtain a sufficiently rich dataset, a minimum of two focus groups were planned, with further groups to be conducted until data saturation was reached, defined as the point at which no new themes emerged on further questioning. Focus groups took place at the postgraduate centre of the host trust, with this being considered the most practical site given that trainees were otherwise dispersed across the region during the week. The recruitment and sampling flow is summarised in Figure 1.

Following permission from the regional Training Programme Director, a recruitment email and participant information sheet were distributed to all eligible trainees by the host postgraduate education department rather than by the lead researcher directly. This step was taken to mitigate recruitment bias and to avoid any sense of obligation that might arise from trainees being approached by a peer. The email emphasised that participation was voluntary and that participants could withdraw at any point up to the time of write-up. A reminder email was planned after four weeks in the event of insufficient responses.

Data collection

Eight trainees consented to participate. They were divided into two focus groups of four. This group size was chosen to make the groups manageable for the moderator and to ensure all participants had the opportunity to respond to each question. Participant characteristics are summarised in Table 2.

**Table 2:** Participant characteristics (n = 8) Characteristics are reported in aggregate to preserve participant anonymity given the single-centre setting. Values are presented as n (%) or median (range).

Characteristic	Values
Training grade	
ST3-ST4	3 (37.5%)
ST5-ST6	3 (37.5%)
ST7+ / post-CCT fellow	2 (25%)
Gender	
Female	5 (62.5%)
Male	3 (37.5%)
Years of postgraduate clinical experience; median (range)	5.5 (3.5-8)
Route into UK training	
UK primary medical qualification	7 (87.5%)
International medical graduate	1 (12.5%)
Working pattern	
Full time	6 (75%)
Less than full time	2 (25%)
Higher degree held or in progress (e.g. MSc, MD, PhD)	
Yes	4 (50%)
No	4 (50%)
Prior formal teaching/education qualification (e.g. PGCert MedEd)	
Yes	1 (12.5%)
No	7 (87.5%)
Approximate grand rounds attended in preceding 12 months; median (range)	20 (18-25)

Each focus group followed a semi-structured format built around five open-ended questions, which were reviewed by medical education peers before use in order to support reliability and validity (see Appendix). Each focus group lasted approximately two hours. No fixed time limit was imposed; sessions continued until data saturation was judged to have been reached. The lead researcher, a neurology specialist trainee in the same region, acted as a facilitator. Where one voice risked dominating the discussion, they actively engaged quieter participants.

All focus groups were audio recorded and the recordings stored on a secure, password-protected drive. Both groups were conducted and transcribed by the lead researcher within seven days, allowing an appreciation of tone and keeping the researcher close to the data.

Data analysis

An inductive conventional qualitative content analysis was undertaken iteratively. Two reviewers--the lead researcher, who was a neurology trainee within the regional programme, and a second reviewer from outside neurology--independently read and re-read each transcript in full. Relevant sections of the transcripts relating to trainees' perceptions of the educational value of the grand round were identified and assigned short descriptive codes.

The reviewers then compared their independently developed codes. Differences in coding or interpretation were discussed until consensus was reached. Related codes were grouped into higher-order categories and, where appropriate, subcategories. For ease of presentation, these higher-order categories are presented as themes and subthemes in the Results section. The developing coding framework was repeatedly compared with the original transcripts to ensure that the analysis remained grounded in participants' accounts and that differing perspectives were retained. All higher-order categories were identified across both focus groups, although participants contributed differing examples and perspectives within each category.

Data saturation was considered during the iterative analytical process. Following analysis of the complete dataset, the reviewers agreed that no new higher-order categories relevant to the research question had emerged, although additional examples and differing perspectives continued to be identified within the existing categories. Data saturation was therefore considered to have been achieved.

The lead researcher's position as a neurology trainee within the regional training programme was recognised as a potential source of researcher bias. To minimise its influence, a second reviewer from outside neurology independently reviewed and coded the transcripts, providing an external perspective and challenging assumptions arising from the lead researcher's familiarity with the grand round setting. Verbatim transcription, independent coding, consensus discussions, iterative comparison with the original transcripts, and consideration of differing perspectives were used to enhance the credibility and trustworthiness of the analysis.

Ethical considerations

Ethical screening was undertaken via the Royal College of Physicians in partnership with UCL, which determined that the study constituted minimal-risk educational research not requiring full research ethics committee review. All participants provided written informed consent, and data were anonymised at the point of collection and stored securely in accordance with General Data Protection Regulation (GDPR) requirements [16].

## Results

Eight neurology trainees participated across two focus groups. Five themes were identified, two of which contained sub-themes. The themes are presented below.

Educational role of consultants

This theme describes how trainees perceived the contribution of consultants to their learning during grand rounds. Participants perceived consultants' expertise during grand rounds to be particularly valuable, especially discussions of clinical reasoning, diagnosis, and management decisions. One trainee commented that “having . . . consultants around makes it really good to learn from them and how they reflect on the case and the management” (P2).

Trainees found differing consultant opinions on management particularly educational, as these discussions reflected the complexity of real-world clinical decision-making. One trainee said, “it was really interesting to see that some consultants would give steroids, but others would go for a biopsy first” (P4). Participants found it particularly insightful when consultants justified their decisions: “Exploring why they would do that is more useful . . . hearing how they would manage it” (P7). Trainees felt that training often focuses heavily on diagnosis, whereas consultant-led discussions during grand rounds shifted attention towards management decisions and practical clinical judgement. Senior trainees especially valued this aspect of grand rounds as preparation for independent practice and several described these discussions as an opportunity to “simulate” consultant-level decision-making.

Not all perceptions were positive. Concerns were raised regarding consultant engagement with teaching, with one participant commenting that “many of the consultants aren’t interested in teaching.” Participants also highlighted the decline in consultant-presented cases, which were viewed as valuable demonstrations of clinical reasoning and presentation style.

Overall, participants described consultant involvement as an important educational aspect of the grand round, while also expressing concerns regarding variable consultant engagement and the decline in consultant-led case presentations.

Questioning style

This theme addresses how the use of questioning by the chairing consultant shaped trainees' perceptions of the session.

Subtheme A: Overly Open Questioning

Participants reported that broad, open-ended questioning could make it difficult to provide focused answers. More specific questioning, such as localisation-based questions, was viewed as more educational and less intimidating; one trainee suggested they could "ask 'where is the lesion?' instead of 'what do you think is going on?'" (P1). Participants proposed mitigating this by using a "generic slide after everything, e.g. what structures are involved, where is the lesion and what do you think the lesion is" (P3). Another participant echoed this by suggesting that the trainees presenting the case could steer the questioning themselves. It was felt that this would help to standardise the questioning process and prevent it from being somewhat of a guessing game, “It should never be a game of, ‘what am I thinking?’ that’s not useful to anyone”(P8). However, opinions differed regarding the extent to which trainees should be driving the questioning, with some participants emphasising increasing independence with seniority.

Subtheme B: Targeting of Individual Trainees

Participants described discomfort when questioning repeatedly focused on the same trainee following an incorrect answer. One trainee commented, “if they don’t know the answers, ask somebody else” (P4). Another participant similarly explained that “there isn't much opportunity to save face in the meeting, for example if you are having an off day” (P1). Participants felt that this reinforced embarrassment and negatively affected the learning environment. Suggestions to improve this included distributing questions more evenly among trainees and involving a broader multidisciplinary audience during discussions.

Overall, participants expressed a preference for more structured questioning and broader distribution of questions among trainees. Broad, open-ended questioning and repeated targeting of individual trainees were described as making participation and learning more difficult.

Learning format

This theme concerns trainees' perceptions of the case-based format of the grand round and how it related to their wider learning. Participants perceived the case-based learning format of the grand round to be an effective teaching format for maximising learning. Trainees stated that “case-based learning is the best format really” (P1), “There is no other way it could be done” (P4).

Participants valued how case-based learning allowed them to apply theoretical knowledge to real clinical cases. One trainee stated that grand rounds “bring to life the textbook” (P3) whilst another felt it “added things which you cannot find in books”. Grand rounds also encouraged reflection on past clinical cases, with one trainee noting, “maybe I could have done that differently!” (P4).

Opinions differed regarding whether trainees should receive information about cases in advance. Some felt prior knowledge improved engagement with more complex learning points, whereas others preferred approaching cases without preparation to better simulate real clinical encounters. Participants also noted that the diverse audience of students, junior doctors, trainees, and consultants could make it difficult to meet all educational needs simultaneously.

Overall, participants described the case-based format of the grand round as a valuable educational approach that helped apply theoretical knowledge and encouraged reflection on previous clinical experiences.

Learning environment and trainee anxiety

Perceptions of the learning environment varied. Some participants described grand rounds as “a supportive environment” (P5) whereas others perceived them as “far too aggressive a forum” (P8).

Participants identified fear of public error and perceived judgement as major sources of anxiety during grand rounds. One participant reflected: “It should be about . . . you’ve said something foolish but its OK because now I know more about it but it doesn’t feel like that at all, it feels like you have literally just exploded any credit you’ve built up by saying something stupid which is a horrible feeling to have!” (P1). Similarly, another trainee explained, “you don’t feel you can make a mistake and learn from it even though that is what it should be about” (P8).

Some participants felt that this anxiety could discourage attendance altogether, with one trainee commenting that “it’s led to some avoidant behaviour from people to not turn up” (P2). However, not all trainees viewed mistakes during grand rounds as particularly consequential, with one participant stating that “What you do in grand round is forgotten two days later” (P4).

Several trainees felt that anxiety negatively affected learning by shifting attention away from the educational content towards fear of embarrassment. One participant reflected, “I have been more bothered about if I am going to be asked the next question than thinking what is actually going on in the case” (P1). Others felt the formal atmosphere discouraged open participation and discussion, “If you know something and you want to answer, you would never put your hand up; I think if it was more relaxed and really educational, you would share your knowledge and ask things you don’t know or understand” (P7). 

**Figure 2:**
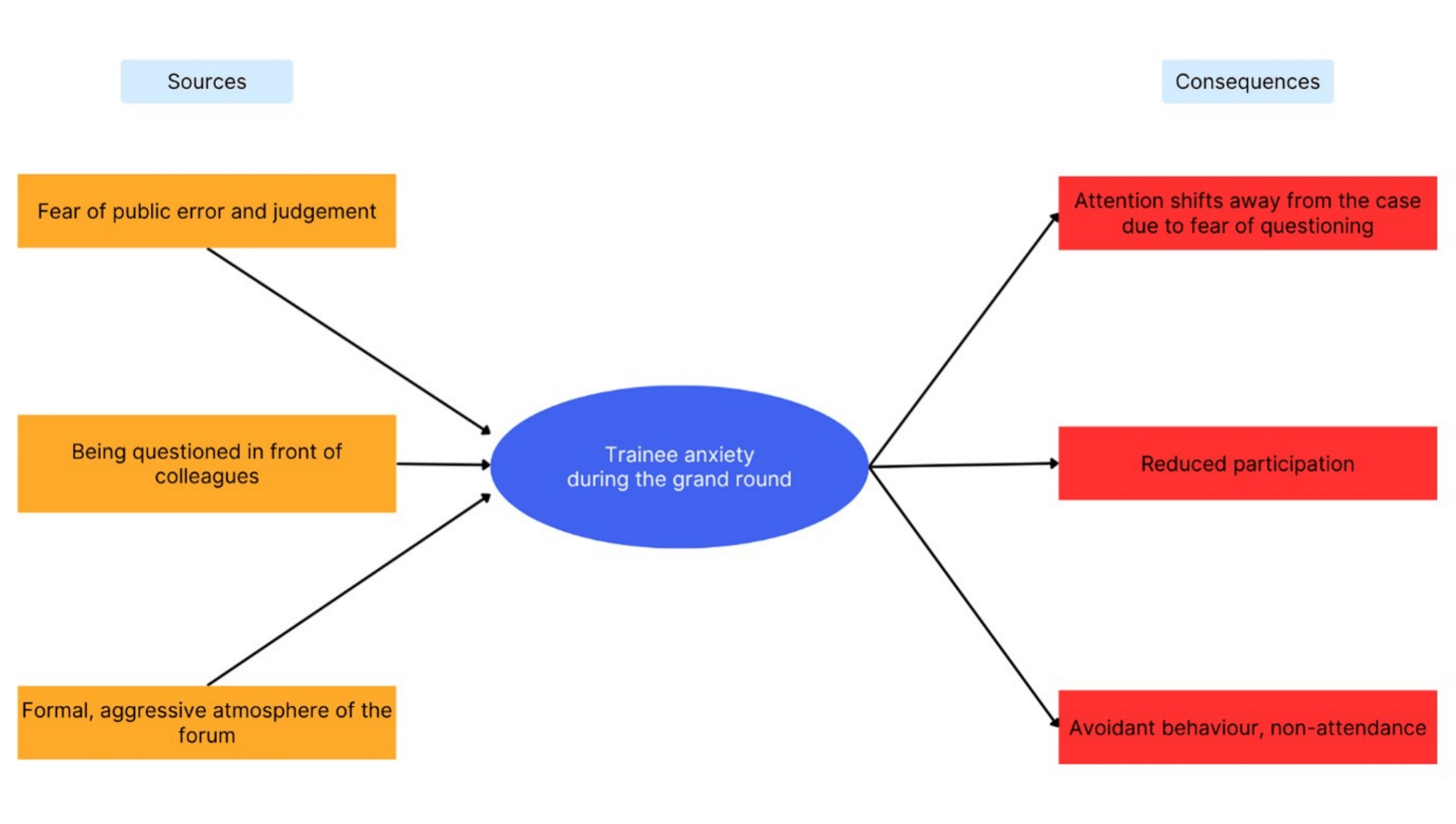
Perceived sources and consequences of trainee anxiety during grand rounds.

Figure 2 was developed from the higher-order categories identified during qualitative content analysis. It visually summarises the perceived sources of trainee anxiety and their educational consequences as reported by participants.

Despite these concerns, some participants recognised educational value in the pressure associated with grand rounds. One participant described it as “a learning experience itself” (P4) that helps trainees “think under pressure and think on your toes” (P4). Another participant similarly reflected, “I like the pressure. I think for me it's OK. But I can appreciate that some people would not like it quite a bit” (P2). Participants also suggested that providing brief case previews beforehand may reduce anxiety and improve engagement with the educational content.

Overall, participants described the grand round learning environment as contributing to anxiety that could adversely affect participation and learning, although a small number also perceived educational value in the pressure associated with the format.

Role of time and experience

This theme describes how trainees perceived their relationship with the grand round format to change as they progressed through training. 

Several trainees reported that grand rounds became more educationally beneficial with increasing seniority and familiarity with the format. One trainee reflected that they “start to enjoy it and actually take in more information the longer you have been doing it” (P4). Participants attributed this to improved prioritisation of clinically relevant information, greater familiarity with colleagues, and reduced anxiety during public discussion. “You learn how to prioritise what is actually clinically relevant . . . as you familiarise yourself more with the people in the room you also become more natural in what you say and less stressed” (P2).

In contrast, junior trainees often described grand rounds as intimidating and difficult to engage with. One participant stated, “being one of the newer people to the system, I spend my whole time just petrified and not taking anything in” (P6). Some participants suggested adaptation strategies for newer trainees, including temporary exemption from questioning during the initial stages of training, “Maybe you should get a free grand round pass for six months” (P4). 

Others felt that early exposure to mistakes may itself be educationally valuable, with one trainee suggesting that “it’s better to make your mistakes early” (P1). Participants also described a sense that senior trainees already possessed greater “credit” with consultants and peers, resulting in reduced concern regarding occasional incorrect answers or uncertainty during questioning.

Table 3 summarises the differences in perceptions between junior and senior trainees identified during qualitative content analysis.

**Table 3:** Differences in grand round perceptions between junior and senior trainees

Junior Trainees	Senior Trainees
Focus on anxiety of the public forum	Focus on transition to consultant
Fear of being made to look ignorant	Comfortable with subject matter and audience
Avoidant behaviour “petrified, not taking anything in”	Value insight into consultant decision making
Little “credit" in the bank to fall back on	Accumulated “credit” reduces stakes
Increasing experience: Anxiety decreases, focus on consultant transition increases	

## Discussion

This study suggests that participants generally perceived the grand round to be an educationally beneficial activity, while identifying several factors that influenced its educational value. In our focus groups, participants located the principal benefits in two areas: the access it gave them to understanding senior decision-making and the capacity for the case-based format to contextualise knowledge. The primary concerns with the neurology grand rounds were noted to be the questioning style employed and the climate of the session.

Importantly, these perceptions were not uniform but varied according to participant seniority, with senior participants reporting lower levels of anxiety associated with the format compared to junior participants. This raises the question of whether a single format may serve junior and senior trainees unequally and whether different formats for trainees at different stages should be considered. Whether this should be in the form of training-stage appropriate questioning, or the consideration of introducing some form of waiver for the initial weeks of training is something which will require more careful deliberation.

Our findings highlight that the grand round is valued, but participants perceived its educational value to vary according to how the session was conducted and where they stood in their training. Set against the wider concern that grand rounds have drifted towards didactic, PowerPoint-based delivery of diminishing value, this study offers an alternative picture. That is, participants perceived the format to retain its educational value, provided that specific concerns were addressed.

Our finding that participants consider case-based discussion as a means of "bringing the textbook to life" is consistent with earlier literature demonstrating that case-based learning promotes learning through contextualisation of prior knowledge [17]. Some trainees, however, did question whether case-based discussions should remain the only organised teaching format. Instead, they argued for additional adjunctive formats of teaching, particularly for the Neurology Specialty Certificate exam (SCE), which is a compulsory written exam taken at the end of training. This is a reservation that mirrors reports in the earlier literature where some learners perceive lecture-based formats as more effective for written examination preparation [18].

Participants perceived grand rounds as providing an opportunity to simulate the clinical decision-making of the consultants who originally encountered the cases presented. This was a particular highlight for many, who expressed that in their current roles they often have limited exposure to making clinical management decisions, with the majority of their time spent in diagnostic work. Participants perceived consultant-level input as providing a valuable opportunity to learn about the clinical management of these often complex neurological cases. This perception is consistent with the literature surrounding expert-level decision making, which emphasises the importance of developing competency in information processing [19]. Furthermore, this insight into consultant-level clinical decision-making will help to ease the transition from registrar to consultant. Difficulties associated with this transition are well recognised within the medical literature [20] and interventions that facilitate this transition have been strongly advocated [21].

Of note, our findings diverged from the literature surrounding questioning styles. Participants perceived broad, unstructured questioning as anxiety-inducing and educationally frustrating, whereas educational theory recognises open-ended questioning as an effective method of assessing higher-order cognition and encouraging movement beyond factual recall towards analysis and synthesis [22]. Our semi-structured interviews suggest that neurology registrars wished to achieve a balance between overly open and closed questioning, perceiving this approach to have greater educational value while avoiding a situation that became a frustrating game of mind reading. This apparent divergence may, in part, reflect the differing educational functions of questioning. While educational theory often discusses open-ended questioning as a strategy for assessing higher-order reasoning [22], participants in our study primarily discussed questioning in terms of its perceived influence on learning and engagement during grand rounds. A related concern was the repeated targeting of specific trainees, which participants perceived as a major source of discomfort and a barrier to learning. Participants described anxiety severe enough to shift attention away from the case and towards fear of public questioning, with some even reporting avoidant non-attendance.

Educational avoidance has been more commonly described in school-aged learners than adult education [23]. With this in mind, one must ask if it is fair or appropriate to continue a practice that induces juvenile avoidant behaviours in clearly capable adult learners. Interestingly, Grieger and Boyd attributed such avoidance behaviours to inappropriate responses to perceived hazards [23]. 

Our findings suggest that participants perceived psychological safety to be important for learning during grand rounds. This perception is coherent with Maslow's hierarchy in which psychological safety underpins motivation and learning [14], with more recent evidence that high-threat public speaking-type environments often impair concentration and memory [24-25]. Nevertheless, our findings also suggest that this relationship is more nuanced. While many trainees perceived anxiety as a barrier to learning, a small number also perceived educational value in the challenge and performance pressure associated with grand rounds, suggesting that an appropriate degree of challenge may be perceived as educationally beneficial by some learners. This observation is consistent with Vygotsky's concept of the Zone of Proximal Development, which proposes that learning is facilitated when learners are appropriately challenged while receiving adequate support [26].

This study has several limitations. Self-selection may have introduced sample bias, as trainees with particularly strong views may have been more likely to participate. This may have influenced the range of perspectives captured, with particularly positive or negative views potentially being overrepresented relative to more neutral experiences. Although the study included the majority of eligible trainees within the regional training programme, the absolute sample size remained small because of the size of the trainee cohort. The findings should therefore be interpreted as an in-depth exploration of trainees' experiences within one regional training programme rather than broadly generalisable conclusions. Furthermore, the study was conducted within a single regional training programme. Grand round format, consultant participation, educational culture, and local training practices may differ between regions and institutions, which may limit the transferability of the findings. Readers should therefore exercise caution when applying these findings to other training programmes, although they may still be relevant to similar educational settings. Although the focus group method generated rich qualitative data, participants may have been reluctant to express controversial or strongly negative opinions in the presence of their peers. The use of an additional questionnaire or individual interviews may have enabled methodological triangulation, although this was not feasible because of time constraints. Additionally, member checking was not undertaken also owing to time constraints, so participants were unable to confirm that the identified categories accurately reflected their intended meaning, and the findings therefore rest on the researchers' interpretation alone, albeit mitigated by independent dual coding and iterative comparison against the transcripts. Furthermore, this study explored trainees' perceptions of the educational value of grand rounds rather than directly measuring learning outcomes. It therefore cannot determine whether the factors identified translated into improved knowledge acquisition, clinical performance, or educational effectiveness. Another limitation relates to the lead researcher being a neurology specialty trainee within the same regional training programme. As an embedded researcher, pre-existing views regarding the educational value of grand rounds may have influenced data collection and analysis despite efforts to maintain an impartial facilitative role throughout the study.

## Conclusions

In conclusion, this study suggests that the neurology grand round remains a valued educational activity for trainees, particularly for case-based discussion, management decision-making and the opportunity to observe consultant-level clinical reasoning. However, several barriers to learning were also identified, including anxiety surrounding the learning environment, overly open questioning styles, and the targeting of individual trainees during public questioning. These findings suggest that trainees perceived trainee experience level, questioning style, and psychological safety to be important influences on the educational value of grand rounds. These factors may therefore warrant consideration in future evaluations of the design and delivery of grand rounds. Given that these findings reflect participants' perceptions from a single regional training programme, similar evaluations in other neuroscience centres would help establish the extent to which these findings are transferable to other neurology training settings. Future research could also explore whether modifications to questioning style or approaches tailored to trainee seniority influence trainees' perceived educational value and engagement during grand rounds.

## Appendices

Appendix

Focus Group Questions

1. What are the benefits of attending the neurology grand round?

2. Are there any barriers to achieving these benefits you mentioned?

3. What do you think about the learning environment in the neurology grand round?

4. What do you think about the use of case-based learning in the neurology grand round?

5. What do you think about having both consultants and registrars attending the neurology grand round together?

## References

[1] (2026). The Complete Guide to Becoming a Neurology Doctor. (2021). Accessed May 23. https://www.bmj.com/careers/article/the-complete-guide-to-becoming-a-neurology-doctor.

[2] Simonds GR, Marvin EA, Apfel LS (2018). Clinical Neuroscience in Practice: An Experiential Learning Course for Undergraduates Offered by Neurosurgeons and Neuroscientists. J Undergrad Neurosci Educ.

[3] Sandal S, Iannuzzi MC, Knohl SJ (2013). Can we make grand rounds "grand" again?. J Grad Med Educ.

[4] Nazari A, Rajesh M, Antoun I, Mohamed Azhar MS, Hayat M (2024). The Student Grand Round: A Peer Teaching Initiative. Cureus.

[5] Goetz CG, Bonduelle M, Gelfand T (1995). Charcot: Constructing Neurology.

[6] Thistlethwaite JE, Davies D, Ekeocha S (2012). The effectiveness of case-based learning in health professional education. A BEME systematic review: BEME Guide No. 23. Med Teach.

[7] Gooding HC, Ziniel S, Touloumtzis C, Pitts S, Goncalves A, Emans J, Burke P (2016). Case-Based Teaching for Interprofessional Postgraduate Trainees in Adolescent Health. J Adolesc Health.

[8] Nordquist J, Sundberg K, Johansson L, Sandelin K, Nordenström J (2012). Case-based learning in surgery: lessons learned. World J Surg.

[9] Van Hoof TJ, Monson RJ, Majdalany GT, Giannotti TE, Meehan TP (2009). A case study of medical grand rounds: are we using effective methods?. Acad Med.

[10] (2026). Socratic Dialogue Gives Way to PowerPoint. (2006). Accessed May 24. https://www.nytimes.com/2006/12/12/health/12docs.html.

[11] Detsky AS (2009). The art of pimping. JAMA.

[12] Kost A, Chen FM (2015). Socrates was not a pimp: changing the paradigm of questioning in medical education. Acad Med.

[13] Knowles MS (1984). Andragogy in Action: Applying Modern Principles of Adult Learning.

[14] Maslow AH (1943). A theory of human motivation. Psychol Rev.

[15] Avis M (2003). Do we need methodological theory to do qualitative research?. Qual Health Res.

[16] (2026). Do I need ethical approval?. https://www.ucl.ac.uk/research-innovation-services/compliance-and-assurance/research-ethics-service/do-i-need-ethical-approval.

[17] Mullins G (1995). The Evaluation of Teaching in a Problem Based Learning Context. Reflections on problem based learning.. Australia.

[18] Kassebaum DK, Averbach RE, Fryer GE Jr (1991). Student preference for a case-based vs. lecture instructional format. J Dent Educ.

[19] Streufert S, Streufert SC: (1978). Behaviour in the Complex Environment..

[20] Morrow G, Illing J, Redfern N, Burford B, Kergon C, Briel R (2009). Are specialist registrars fully prepared for the role of the consultant?. Clin Teach.

[21] Dean A (2003). Mentors for newly appointed consultants. Adv Psychiatr Treat.

[22] Hift RJ (2014). Should essays and other "open-ended"-type questions retain a place in written summative assessment in clinical medicine?. BMC Med Educ.

[23] Grieger RM, Boyd JD (2006). Childhood Anxieties, Fears, and Phobias: A Cognitive-Behavioral, Psychosituational Approach. Rational Emotive Behavioral Approaches to Childhood Disorders. Rational emotive behavioral approaches to childhood disorders: Theory, practice and research.

[24] Wenzel A, Holt CS (2003). Social-evaluative threat and cognitive performance in socially anxious and non-anxious individuals. Pers Individ Dif.

[25] Fox E, Russo R, Bowles R, Dutton K (2001). Do threatening stimuli draw or hold visual attention in subclinical anxiety?. J Exp Psychol Gen.

[26] Vygotsky LS (1978). Mind in Society: The Development of Higher Psychological Processes.

